# Sleep disturbance in patients taking opioid medication for chronic back pain

**DOI:** 10.1111/anae.13601

**Published:** 2016-08-22

**Authors:** J. A. Robertson, R. J. Purple, P. Cole, Z. Zaiwalla, K. Wulff, K. T. S. Pattinson

**Affiliations:** ^1^ Nuffield Department of Clinical NeurosciencesUniversity of OxfordJohn Radcliffe HospitalOxfordUK; ^2^ Nuffield Department of Clinical NeurosciencesUniversity of OxfordOxford Molecular Pathology InstituteSir William Dunn School of PathologyOxfordUK; ^3^ Department of NeuroscienceJohn Radcliffe HospitalOxfordUK

**Keywords:** chronic opioids: side effects, chronic pain, sleep disturbance

## Abstract

Poor sleep is an increasingly recognised problem with chronic pain and further increases the effect on daily function. To identify the relationship between chronic pain, opioid analgesia and sleep quality, this study investigated activity and sleep patterns in patients taking opioid and non‐opioid analgesia for chronic back pain. Thirty‐one participants (10 healthy controls, 21 patients with chronic pain: 6 on non‐opioid medication; 15 on opioid medication) were assessed using actigraphy, polysomnography and questionnaires. Patients with chronic pain subjectively reported significant sleep and wake disturbances as shown by decreased overall sleep quality (Pittsburgh Sleep Quality Index, p < 0.001), increased symptoms of insomnia (Insomnia Severity Index, p < 0.001) and increased fatigue (Fatigue Severity Scale, p = 0.002). They also spent increased time in bed (p = 0.016), took longer to get to sleep (p = 0.005) and had high interindividual variability in other measures of activity but no overall irregular rest‐activity pattern. Patients on high doses of opioids (> 100 mg morphine‐equivalent/day) demonstrated distinctly abnormal brain activity during sleep suggesting that polysomnography is necessary to detect sleep disturbance in this population in the absence of irregular rest‐activity behaviour. Night‐time sleep disturbance is common in individuals suffering from chronic pain and may be further exacerbated by opioid treatment. Considerations must be made regarding the appropriate use of combined actigraphy and miniaturised polysomnography for future population‐based studies.

## Abstract 2

☛ CPD available at http://www.learnataagbi.org

## Introduction

Sleep disruption has been associated with a wide range of health problems including reduced daily function 1, increased pain 2, psychiatric disorders 3 and type‐2 diabetes 4. It is also a common problem in patients with chronic pain 5, 6 with one study reporting 89% of 287 chronic pain patients experiencing disrupted sleep 2. The prevalence of chronic pain has been estimated to be 19% in European adults and up to 41% in low‐income countries 7, 8. Opioid medication, estimated to be prescribed to 28% of patients with chronic pain in Europe 7, has been shown to decrease deep sleep (stages 3 and 4) and increase stage 2 sleep on acute administration in healthy adults 9, 10, 11, and may cause instability in deep sleep, increased waking and decreased rapid eye movements (REM) with chronic use in addicts 12. Additionally, opioid‐induced respiratory depression and sleep‐disordered breathing may exacerbate this sleep disruption. One study found 85% of patients taking opioid medication (median morphine‐equivalent dose: 180 mg.day ^−1^ [range: 7.5–935 mg]) suffer from some form of sleep apnoea 13, and increases in central apnoea and decreased arterial oxygen saturation have been demonstrated in patients taking opioid medication 14, 15, 16. Together, the evidence highlights a considerable problem with sleep disruption in chronic pain, which may be particularly severe in patients taking opioid medication.

Sleep can be assessed both subjectively, using sleep diaries and questionnaires, and objectively, using polysomnography. While subjective questionnaires give an insight into patients' experiences of pain and sleep disturbance, objective polysomnography allows a more direct assessment of physiological differences. Actigraphy is a useful additional measure to monitor rest/activity behaviour to detect abnormal circadian and ultradian patterns. Discrepancies between objective and subjective measurements of sleep may be common 17, 18. For example, insomnia sufferers have been found to report shorter sleep duration than healthy controls with a similar objective sleep length 18, suggesting an impact of sleep perception on overall quality. Therefore, to adequately assess the impact of sleep disruption on an individual, both objective and subjective measurements are necessary. Actigraphy uses long‐term monitoring of the level of activity from arm movement as a proxy for wake rest. Sleep‐wake timing and movement‐related sleep parameters are then inferred from this data. As an inexpensive and minimally disruptive device, actigraphy has the potential to be used for large population‐based studies, enabling researchers to gather rest‐activity data over lengthy periods of time but not cortical sleep or breathing data. Polysomnography, with overnight electroencephalography derived from electrical brain activity and additional physiological measurements, allows determination of sleep stages and structure as correlates of sleep in addition to measurements of respiratory events during sleep. While this gives very detailed information about sleep, it is time and resource intense.

Little research has directly addressed the question of how opioid medication may affect subjective and objective sleep in chronic pain patients. Actigraphy studies have shown that people with chronic pain spend more time in bed, have a lower sleep efficiency than healthy individuals and lower number of movement‐related awakenings after sleep onset 19, 20. One study showed that individuals with prescription opioid dependence had significantly disturbed sleep on actigraphy in addition to subjective poor sleep quality identified by the Pittsburgh Sleep Quality Index 21. Polysomnography studies have identified sleep‐disordered breathing in patients on chronic opioid therapy 16, 22.

This study included an evaluation of the feasibility of actigraphy and polysomnography as techniques to assess rest‐activity timing and physiological sleep, in a population of patients with chronic pain. From this, the study also aimed to allow the generation of future hypotheses regarding the effects of opioid medication on sleep which could be formally tested in population‐based studies.

## Methods

Ethical approval was obtained from the Oxford NHS Research Ethics Committee and all participants provided written informed consent. The study included 31 participants (10 healthy controls, 21 patients with chronic pain: 6 on non‐opioid medication; 15 on opioid medication). The sample size was chosen to provide preliminary data for a larger subsequent study and was estimated to be sufficient to provide an understanding of any issues that running a larger study may entail. Participants in both patient groups were between the ages of 18–65 and taking opioid or non‐opioid medication for chronic back pain. The duration of opioid medication was 1–35 years (mean (SD) 7.5 (8.6) years). Pain medications taken by patients in the non‐opioid group included paracetamol, ibuprofen, amitriptyline and diclofenac. Exclusion criteria included any significant cardiac, respiratory, neurological or metabolic disease; evidence or history of substance misuse; travel across more than two time zones in the previous month or travel across one time zone in the previous week; and shift work. Inclusion criteria for healthy controls included no significant co‐morbidity and not taking any opioid, antidepressant or sleep medication.

All participants completed six questionnaires in order to assess their subjective impression of their sleep and pain: the Pittsburgh Sleep Quality Index (PSQI, a composite scale with a range of 0–21, where a higher score indicates a worse sleep quality) 23; Insomnia Severity Index (ISI, a subjective assessment of sleep with a score range of 0–28. A higher score indicates increasing trouble with sleep) 24; Pain Vigilance and Awareness Questionnaire (PVAQ, a score of pain‐related experiences with a score range of 0–80 with a higher score indicating increased pain vigilance and awareness) 25; Centre for Epidemiological Studies Depression Scale (C‐ESD, a score with a range of 0–60, with higher scores indicating increasing psychological distress) 26; the Epworth Sleepiness Scale (ESS, an evaluation of daytime sleepiness with a score range of 0–24, with a higher score indicating increased daytime sleepiness) 27; and the Fatigue Severity Scale (FSS, a score to assess the impact of fatigue on daily living with a range of 9–63 with a higher score indicating a greater impact of fatigue on aspects of daily life.) 28. Full questionnaire details and references are available in the Appendix All participants wore an actiwatch (Actiwatch‐L, CamNTech Ltd., Cambridge, UK) for 14 continuous days and kept a sleep and activity diary, documenting bed‐time, get‐up time, naps, prolonged periods of rest (e.g. watching television) and any times the actiwatch was removed (e.g. for swimming), during this period. Actigraphy data were analysed with ‘Actiwatch Activity and Sleep Analysis’ software (CamNtech Motionware version 1.1.3; Cambridge Neurotechnology, Cambridge, UK). Data from the sleep diary were used to annotate the actigraphy data and to edit accordingly. Actigraphically‐derived sleep parameters such as sleep timing, sleep efficiency and sleep latency, fragmentation index, interday stability (consistency of movement patterns over many days) and intraday variability (structural similarity of movements within 24 h) were calculated using the automated algorithms within the software. A subset of 12 participants underwent two consecutive nights of domiciliary polysomnography recording (SomnoScreen+PSG, SOMNOmedics GmbH, Randersacker, Germany). This recorded electrical activity in the brain (electroencephalography, EEG), muscle activity (electromyography, EMG), and eye movements (electrooculography, EOG), in addition to physiological measurements of heart rate, oxygen saturation of arterial blood, respiratory effort and flow recordings. For the EEG, a standard montage of five channels (Fz, Cz, C3, C4, Oz) referenced to the mastoids was used, sampled at 128 Hz and digitally filtered using finite impulse response (FIR) band pass filtering between 0.2 Hz and 35 Hz with the Hamming window. All data were collected in the participants' home environment and all were encouraged to follow their usual daily routines. The first night acted as an adaptation night, and data were extracted and analysed from the second night's recording 29. Polysomnography data were analysed using the DOMINO version 2.6.0 software (SOMNOmedics GmbH) and scored according to the American Academy of Sleep Medicine criteria with the separation of both stage 3 and stage 4 sleep to gain greater macrostructural detail (AASM Manual for Scoring Sleep, 2007).

For group analysis, patients taking opioid medication were divided into higher (~100 mg morphine‐equivalent.d ^−1^) and lower dose (< 100 mg morphine‐equivalent/day) groups as it has been demonstrated that > 100 mg may be a high‐risk dose 30. The daily morphine‐equivalent dose was calculated using a published reference 31. All data were analysed using SPSS (IBM Corp. Released 2012. IBM SPSS Statistics for Macintosh, Version 21.0; Armonk, NY: IBM Corp). Questionnaire results were analysed with the Mann–Whitney U‐test, adjusted for multiple comparisons using a Bonferroni corrected significance value of α = 0.0056. Relevant actigraphic parameters were compared with the Mann–Whitney U‐test with a significance value of α = 0.05. Polysomnography data were similarly analysed with the Mann–Whitney U‐test and generalised linear models for count and proportion data. Finally, a regression analysis was carried out to compare questionnaire and actigraphic parameters with opioid dose. The primary outcome measures (actigraphic sleep fragmentation, sleep efficiency and opioid dosage) for one participant was three standard deviations above the mean and was therefore removed from this analysis due to a statistical relationship that was unable to be confirmed from this small sample size (n = 14) 32. Please see the appendix for regression analysis and plots with and without the outlier.

## Results

Thirty‐one participants (10 healthy controls, 21 patients with chronic pain) took part in the study. Compliance with questionnaires, actigraphy and sleep diaries was very good with few cases of incomplete data due to not keeping the actiwatch on or inaccurate diary data. Polysomnography, particularly after a trial night, was also well‐tolerated, allowing participants to sleep in their own, natural environment. The characteristics of participants in each group are shown in Table 1.

**Table 1:** Sleep and rest‐activity characteristics of study participants. Values are mean (SD)

	Participant group (medians [IQR])				p value, patients vs. controls	p value, high‐dose vs. non‐opioid
	High‐dose opioid medication n = 4	Low‐dose opioid medication n = 11	Non‐opioid medication n = 6	Healthy controls n = 10		
Group characteristics						
Age; years	42 (28)	44 (22)	44 (28)	44 (27)	–	–
Morphine‐equivalent.d^−1^; mg	116.5 (89.5)	10 (68.9)	0	0	–	–
Number of men	n = 2	n = 4	n = 3	n = 6		
Subjective sleep, pain and mood						
Sleep quality (PSQI)	10.5 (3.3)	15.0 (7.0)	11.0 (8.0)	3.0 (2.5)	<0.001	0.915
Insomnia symptoms (ISI)	11 (2)	18 (10)	16 (15)	2 (3)	<0.001	0.915
Daytime sleepiness (ESS)	15.0 (6.6)	7 (11.5)	6.0 (11.1)	4.5 (7.8)	0.056	0.114
Fatigue (FSS)	43.5 (21.5)	36.0 (13.5)	55.0 (25.3)	20.5 (10.3)	0.002	0.669
Pain rating (PVAQ)	56.0 (28.8)	49.0 (20.0)	51.0 (32.0)	32.5 (16.3)	<0.001	0.762
Depressive symptoms (CESD)	22.0 (17.0)	12.0 (23.0)	12.0 (15.0)	5.5 (6.3)	0.012	0.392
Actigraphic sleep parameters						
Time in bed; h	10.0 (2.1)	8.7 (1.6)	9.4 (2.0)	8.0 (1.4)	0.016	0.257
Total sleep time; h	7.9 (2.5)	7.3 (1.0)	6.9 (1.9)	6.4 (1.0)	0.026	0.610
Sleep efficiency; %	81.9 (24.7)	87.6 (3.3)	82.1 (10.2)	83.8 (4.3)	0.808	1
Sleep latency; min	30.0 (44.2)	24.9 (20.5)	23.2 (21.9)	11.0 (8.6)	0.005	0.476
Fragmentation index	37.1 (71.0)	29.6 (11.0)	36.1 (16.2)	34.3 (13.0)	0.725	0.914
Interdaily stability	0.6 (0.2)	0.5 (0.2)	0.5 (0.2)	0.3 (0.2)	0.059	0.521
Intradaily variability	0.8 (0.6)	0.8 (0.3)	1.1 (0.3)	1.0 (0.1)	0.484	0.669
Polysomnographic sleep parameters						
Time in bed; h	8.2 (1.1)	–	8.3 (1.8)	7.4 (0.5)	0.154	1
Total sleep time; h	7.0 (1.5)	–	7.6 (2.2)	6.8 (0.8)	0.461	1
Sleep efficiency; %	90.2 (12.8)	–	93.0 (2.9)	95.1 (4.9)	0.367	0.794
Stage 1 (%TST)	13.7 (3.6)	–	17.7 (7.6)	17.8 (7.0)	0.924	0.756
Stage 2 (%TST)	61.6 (30.9)	–	40.1 (10.1)	40.5 (11.8)	0.303	0.595
Stage 3 (%TST)	7.0 (7.4)	–	11.3 (3.3)	11.6 (2.7)	0.707	0.913
Stage 4 (%TST)	5.8 (11.3)	–	7.4 (7.6)	6.2 (6.2)	0.551	0.902
REM sleep (%TST)	11.9 (15.7)	–	15.6 (11.4)	20.2 (3.6)	0.276	0.815
Frequency of OSA	19.0 (135.0)	–	0.5 (1.8)	2.0 (2.5)	0.575	0.491
Frequency of CSA	0.5 (5.8)	–	4.0 (3.8)	0.0 (0.5)	0.267	0.108
Respiratory rate	10.0 (2.3)	–	12.5 (2.5)	15 (3.5)	0.170	0.144

For questionnaires an adjusted Bonferroni significance value of α = 0.0056 was used.

M, male; F, female; PSQI, Pittsburgh Sleep Quality Index; ESS, Epworth Sleepiness Scale; ISI, Insomnia Severity Index; FSS, Fatigue Severity Scale; PVAQ, pain vigilance and awareness questionnaire; C‐ESD, Center for Epidemiological Studies Depression Scale; TST, total sleep time; OSA, obstructive sleep apnoeas; CSA, central sleep apnoeas.

**Figure 1:**
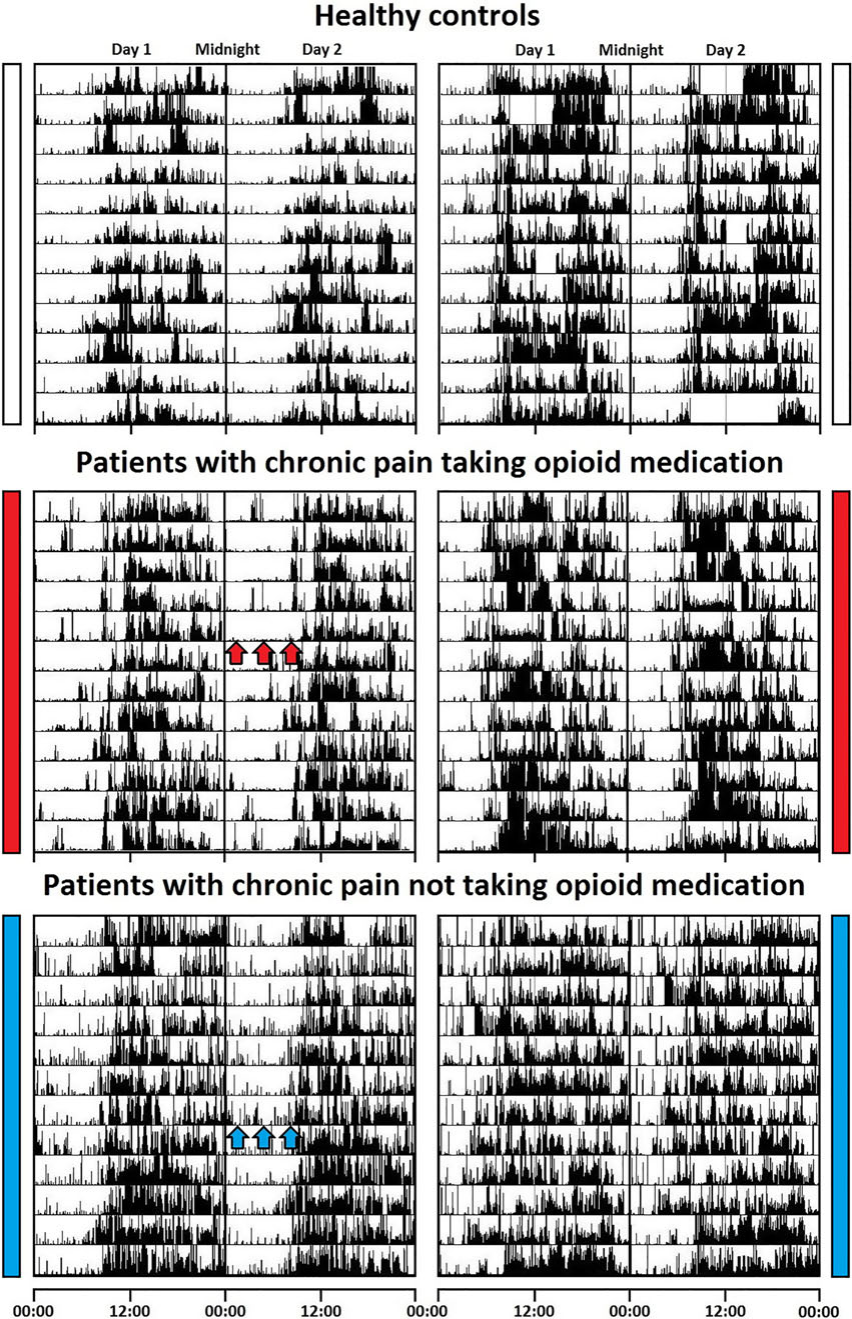
Example actigraphy traces of 12 consecutive days from two controls, two patients with chronic pain who take opioid medication and two patients with chronic pain but on treatment other than opioid medications. Black bars indicate activity, with the same scale used for each actogram. Each actogram is 48 h double‐plotted with successive days on the vertical axis. The midline of each actogram represents midnight between days 1 and 2. In this sample, actigraphy shows continuous activity during the night in patients not on opioid medication (blue bar/arrows) while patients on opioid medication are least active during the night (red bar/arrows). Top left, 62‐year‐old female, top right, 44‐year‐old male. Middle left, 52‐year‐old female morphine equivalent dose = 210 mg, middle right 41‐year‐old male morphine equivalent dose = 71 mg, bottom left, 51‐year‐old female, bottom right 51‐year‐old male. (Opioid dose expressed as morphine equivalents per day).

**Figure 2:**
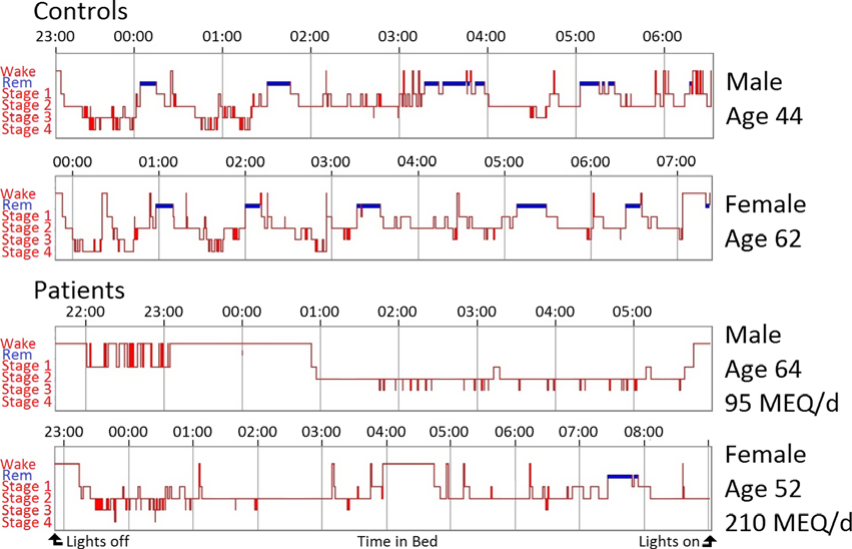
Top, hypnograms from two healthy controls presenting characteristic electroencephalographic correlates of sleep and its architecture with approximate 90‐min sleep cycles transitioning from wake to NREM light sleep, NREM deep sleep and finally REM sleep. Bottom, hypnograms from two patients taking high doses of opioids (expressed as morphine equivalents per day, MEQ.d^−1^). Here, abnormal sleep architecture is clearly demonstrated with a large proportion of the night awake or in stage 2 sleep, with striking deficits in REM and deep sleep. The horizontal axis begins at lights off and ends at lights on, with the length dependent upon how long the individual tries to sleep for. The vertical axis corresponds to vigilance state (Wake, REM: Rapid Eye Movement sleep; Stage 1: light sleep; Stage 2: durable sleep; Stage 3: transitional sleep toward deep sleep; Stage 4: deep sleep).

Taking into account all participants taking opioid medication, with the exception of an outlier (n = 13), linear regression revealed no relationship between opioid dosage and pain rating, subjective sleep quality (PSQI), or any actigraphic measurement (adjusted R‐squared −0.14, p value 0.69). Representative actograms and EEG hypnograms of participants from each group are shown in Figs. 1 and 2, respectively. Several patients experiencing chronic pain had severely fragmented sleep compared with healthy controls (Fig. 1). Polysomnographic sleep assessment revealed that two out of the four patients taking > 100 mg morphine‐equivalent.d ^−1^ had extremely abnormal hypnograms with deficits in deep sleep, REM sleep, decreased respiratory rate and a high frequency of obstructive and central sleep apnoea compared with all other participants.

## Discussion

To the authors' knowledge, this is the first study to directly contrast subjective and objective sleep parameters with the use of questionnaires, actigraphy and polysomnography in patients taking opioid medication for chronic pain, patients not taking opioid medication for chronic pain and healthy controls. Substantial sleep disruption was identified in patients experiencing chronic pain, which was primarily revealed through subjective measures, although objective actigraphy indicated longer sleep onset latency and greater time in bed. No differences in subjective sleep measures or actigraphic parameters were found between patients taking high doses of opioid medication, and patients not taking opioid medication. However, analysis of individual data demonstrated markedly abnormal polysomnographic sleep architecture and increased sleep‐disordered breathing in two out of four opioid users. These findings suggest that chronic pain can be associated with significant disruption in brain activity during sleep. Furthermore, this disruption is not improved, and may even be exacerbated, in patients taking opioid medication.

Subjective parameters revealed that patients experiencing chronic pain had decreased sleep quality (PSQI), increased symptoms of insomnia (ISI), increased fatigue (FSS) and a trend towards increased daytime sleepiness (ESS). In particular, daytime sleepiness was increased in the high‐dose opioid group. Although this increase was not significant, the median score (15) was greater than the upper threshold (10) for ‘normal’ ESS values 33. This indicates that the longer time to sleep onset and longer time in bed as revealed by actigraphic measures may be due to increased sedation that may not have been restful and induced daytime sleepiness. No differences were found in the fragmentation index or sleep efficiency, two primary measures of sleep disruption, which might be explained by high variability within groups and a small sample size, or masking due to medication side‐effects. Previous research using actigraphy in patients with chronic pain has been largely inconclusive. A study in older persons with chronic pain also identified worse PSQI sleep quality scores but no difference in daytime sleepiness using the Epworth Rating Scale 19. Actigraphically derived time in bed was longer but no differences were found for total sleep time, sleep efficiency or sleep onset latency. Patients with chronic lower back pain were found to have significantly worse PSQI sleep quality scores and lower actigraphic sleep efficiency but no differences in actigraphic sleep onset latency or total sleep time 20. Inconsistencies likely arise across the literature due to variable population groups with heterogeneous primary pain symptoms, medication use, age and methodological differences including recording length, sampling interval and scoring algorithm used. The longer total sleep time of patients in our study may be explained by the sedative effects of medication, and by the rather heterogeneous control group.

Poor sleep quality (PSQI > 5) was identified in 93% of individuals taking opioid medication compared with 20% of the control group and the Epworth sleepiness scale revealed that five of six participants rated as ‘excessively sleepy during the day’ were taking opioid medication. Overall, no differences were found in terms of any subjective or objective sleep parameters for patients taking high doses of opioids compared with those not taking opioids. Further, no association was found with any sleep PSG or actigraphic parameters and increasing daily opioid dose. This finding is consistent with previous research revealing no effect of opioid medication on polysomnographically determined total sleep time, sleep efficiency, sleep latency or awakenings in healthy individuals when taken acutely (with the effect of chronic use largely unstudied) 9, 10. However, we appreciate that the small sample size and preliminary nature of this study means that subtle effects of opioids may have been missed.

**Figure 3:**
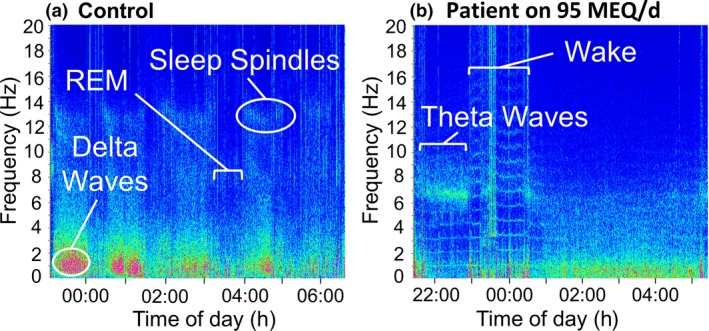
Overnight spectral fast Fourier transform (FFT) of the central C4 electroencephalography channel. (a) healthy control. Patterns are well matched to models of human sleep architecture. Periodic delta frequency bands (0.5–2 Hz) representing deep sleep (stage 3 and 4) are clearly identified in pink, presenting greatest intensity from the beginning of the night and gradually decreasing towards the end. Sigma frequency bands can also be identified (in the range 11–14 Hz) representing sleep spindles of stage 2 sleep. Four bouts of REM sleep can be identified with increasing duration, represented by periods of low intensity and a lack of delta frequencies. (b) Participant on 95 mg of opioids for chronic back pain. In contrast to the healthy control, no discernible pattern of sleep architecture is present. Instead of delta waves, this individual shows theta waves characteristic for stage 2 sleep. Both sigma and delta frequencies are nearly absent, suggesting this individual has abnormal sleep spindles and does not enter deep sleep. Furthermore, there is an absence of REM sleep, with this individual likely alternating between wake, stage 1 and stage 2.

The effect of opioid medication on brain activity in terms of sleep architecture is particularly interesting and was only identified through polysomnographic measures. Due to the resource‐intense nature of polysomnography, data collection for patients taking opioid medication was limited to patients taking high doses. Examination of individual patient data demonstrated markedly abnormal sleep architecture with reduced REM, reduced stage 3/4 and longer stage 2 sleep in two out of four patients taking high doses of opioid medication in line with other polysomnographic studies on opioid use (Figs. 2 and 3) 9, 10. In healthy individuals, stage 2 sleep comprises around 45% of total sleep time, with REM sleep and deep sleep (stage 3/4) each comprising around 25%, which is similar to our controls and patients not taking opioids (Table 1) 34. However, patients taking high doses of opioid medication spent only about 12.5% of time in REM and stage 3/4 NREM sleep each, while stage 2 NREM sleep increased to 60% of total sleep time, most likely as a compensation for the inability to enter stage 3/4 NREM and REM sleep. Previous studies have revealed a significantly longer period in stage 2 sleep, a shorter period in deep sleep and some evidence for shorter REM sleep after acute administration of opioid medication in healthy volunteers, which is supported through our findings, suggesting a continued negative effect with chronic use 9, 10, 11. Furthermore, the same two patients taking high doses of opioid medication showed a lower respiratory rate and a considerably greater number of central and obstructive apnoeas (one night of PSG identified 57 apnoeas in one patient and 432 in the other compared with < 15 in all other participants). This follows previously established evidence for respiratory depression during sleep from opioid medication and could contribute to additional sleep disruption in this group 15, 35, 36. Interestingly, in this sample the majority of this increase originated from increased obstructive sleep apnoea (OSA, high‐dose opioids, median frequency 19) as opposed to central sleep apnoea (CSA, high‐dose opioids, median frequency 0.5). This may reflect the comorbidities which are often present in patients with lower back pain and a larger sample size would likely elucidate this. Nevertheless, if left untreated, patients with OSA have been shown to develop cognitive impairment and dementia of Alzheimer's type about 10 years earlier than those without OSA or being treated with continuous positive airway pressure 37.

In considering the clinical applications of these methods, our findings suggest that, while actigraphy facilitates collection of data over considerable stretches of time, it should be paired with polysomnography and subjective sleep assessments in order to give a more comprehensive understanding. This may be particularly relevant in situations such as intensive care, where simple environmental interventions have been shown to improve subjective sleep quality and reduce the odds of developing delirium 38, 39. Here, further in‐depth investigations may elucidate the extent to which quality of sleep (as assessed objectively by polysomnography and actigraphy) can be improved by such interventions. Both actigraphy and polysomnography could be easily used in an intensive care setting 40, with minimal disruption or discomfort to the patient, and the use of polysomnography in addition to actigraphy would be especially important given that patients in intensive care are likely to be sedated. Furthermore, the addition of the recording of respiratory parameters and pulse oximetry in polysomnography could facilitate assessment of sleep apnoea in postoperative settings, allowing enhanced understanding of the association of obstructive sleep apnoea with adverse peri‐operative outcomes 41.

Although the long‐term effect of specific sleep stage deficit is largely unknown, sleep disruption is likely to have significant impact on health through suppression of its major functions, that could disrupt memory consolidation, immune function and cognitive performance 42. The implications of these findings for patient quality of life poses new considerations for the use of opioid medication and the best strategy for treatment of chronic pain with the minimisation of detrimental effects on sleep. Future studies are needed to investigate these effects of opioid medication on brain activity with particular emphasis on high dose use in addition to possible differential effects between opioid medications.

There are limitations to the conclusions that can be drawn from this study, including the small sample size and large range of ages included (given that age can affect sleep and sleep structure 43). Furthermore, it is difficult in these groups of patients to disentangle the effects of pain and pain medication on sleep, in addition to the potential impact of social circumstances. These issues may account for the lower than expected sleep efficiency and increased stage 1 sleep in the control group represented in both EEG and actigraphy 44. Future studies should, therefore, use larger sample sizes with more uniform age ranges or grouped according to age.

Actigraphy is not designed to detect brain activity and it cannot be used to distinguish between periods of motionless wakefulness and a person asleep, which may be common among this population due to a degree of sedation caused by medication 45. These limitations may also apply to the use of applications on domestic portable electronic devices (e.g. ‘apps’ on smart phones) which monitor sleep using accelerometers. Such software is becoming commonplace. The EEG/polysomnography records cortical frequencies but is time‐intensive and, therefore, was only used in a small subset of participants and over two nights in this study. Despite this, polysomnography revealed highly abnormal brain activity during sleep, and our study suggests that it is currently the most sensitive tool to objectively describe the sleep disruption due to pain and medication in at‐home settings. Our study also suggests that questionnaires are vital for evaluating the perceived sleep quality and the impact of its disruption on quality of life with chronic pain.

Overall, this study has shown that sleep disruption is common but heterogeneous in patients with chronic pain being treated with opioid as well as non‐opioid medication. Profiling the various types of sleep disruption in patients with chronic pain required three different methods; questionnaires; actigraphy; and polysomnography. These data have demonstrated the utility of subjective and objective measures in the assessment of sleep in pain and have highlighted the potential for opioid medications to interfere with sleep regulation. It would, therefore, benefit future studies to take these factors into account when interpreting findings and a requirement for several measures of sleep quality would be optimal.

## Competing interests

No competing interests declared.

## Figures and Tables

**Figure A1:**
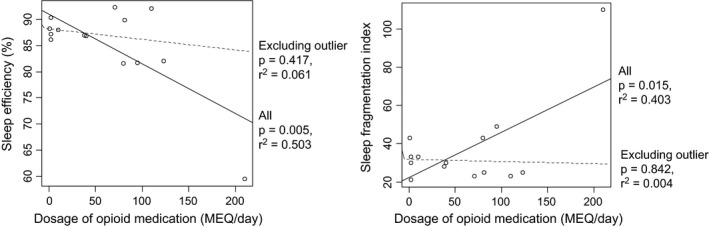
Effect of daily dosage of opioid medication (morphine equivalent, MEQ) on average actigraphic sleep efficiency (left) and sleep fragmentation (right), including and excluding outlier data (n = 14 and n = 13, respectively).

## Appendix 1 Questionnaire scoring reference

### Pittsburgh Sleep Quality Index (PSQI) 23

The PSQI assesses seven components of sleep including: subjective sleep quality; sleep latency; sleep duration; habitual sleep efficiency; sleep disturbances; use of sleeping medication; and daytime dysfunction. Nineteen self‐related questions are answered to produce a score for each component (0 = no difficulty, 3 = severe difficulty). The global PSQI score is calculated by the sum of the component scores with a range of 0–21, with a higher score indicating a worse subjective sleep quality (for a full description of the scoring method see Buysse et al. 23). A cut‐off score of 5 provides a sensitivity of 89.6% and a specificity of 86.5% 23 for identifying patients with poor sleep and is widely used.

### Insomnia Severity Index (ISI) 24

The ISI consists of seven questions that require you to rate subjective aspects of sleep. The scores are added together to provide a composite score (range = 0–28). Score categories are: 0–7 = no clinically significant insomnia; 8–14 = subthreshold insomnia; 15–21 = Clinical insomnia (moderate severity); 22–28 = Clinical insomnia (severe).

### Pain Vigilance and Awareness Questionnaire (PVAQ) 25

The PVAQ contains 16 items relating to the subjects pain‐related vigilance and awareness over the last 2 weeks, each rated by the subject from 0 (never) to 5 (always) with regard to how frequently each item was a true description of them. A composite score is then calculated, with two items ‘reverse keyed’ 25. A higher score indicates increased pain vigilance and awareness.

### Centre for Epidemiological Studies Depression Scale (C‐ESD) 26

The C‐ESD is designed to measure ‘depressive symptomatology in the general population’. The score consists of 20 items and the subject is required to rate how often each thought or symptom was present. The range of scores are 0–60, with a higher score indicating increased psychological distress, and scores > 16 suggesting a clinically significant level of psychological distress.

### The Epworth Sleepiness Scale (ESS) 27

The ESS aims to evaluate daytime sleepiness by asking subjects to score how likely they would be to fall asleep in eight different, varyingly soporific situations. Scores range between 0 and 24, with a higher score indicating increased daytime sleepiness. A score of over 16 indicates high levels of daytime sleepiness compared to the general population.

### Fatigue Severity Scale (FSS) 28

The FSS assesses the impact of fatigue on daily living with a score range of 9–63. It consists of 9 statements for which patients select a number from 1 to 7 to indicate the extent to which they agree or disagree (1 = strongly disagree, 7 = strongly agree). A higher score indicates a greater impact of fatigue on aspects of daily life.

### Regression analysis

Taking into account all participants on opioid medication (n = 14), linear regression revealed that a higher amount of daily opioid dose was associated with increased actigraphic sleep fragmentation and lower actigraphic sleep efficiency (p = 0.008, p = 0.002 respectively, Fig. A1). Linear regression also revealed that increased subjective pain rating (PVAQ) was associated with lower actigraphic sleep fragmentation and greater actigraphic sleep efficiency (p = 0.01, p = 0.017 respectively, not shown). With the exclusion of an outlier (3 standard deviations above mean), all linear regression results became non‐significant (p > 0.05). No relationship was found between opioid dosage and pain rating, subjective sleep quality (PSQI), or any other actigraphic measurement.
